# Salicylic Acid Enhances Adventitious Root and Aerenchyma Formation in Wheat under Waterlogged Conditions

**DOI:** 10.3390/ijms23031243

**Published:** 2022-01-23

**Authors:** Murali Krishna Koramutla, Pham Anh Tuan, Belay T. Ayele

**Affiliations:** Department of Plant Science, University of Manitoba, 222 Agriculture Building, Winnipeg, MB R3T 2N2, Canada; murali.koramutla@umanitoba.ca (M.K.K.); anh.pham@umanitoba.ca (P.A.T.)

**Keywords:** adventitious roots, aerenchyma, salicylic acid, waterlogging, wheat

## Abstract

The present study investigated the role of salicylic acid (SA) in regulating morpho-anatomical adaptive responses of a wheat plant to waterlogging. Our pharmacological study showed that treatment of waterlogged wheat plants with exogenous SA promotes the formation axile roots and surface adventitious roots that originate from basal stem nodes, but inhibits their elongation, leading to the formation of a shallow root system. The treatment also enhanced axile root formation in non-waterlogged plants but with only slight reductions in their length and branch root formation. Exogenous SA enhanced the formation of root aerenchyma, a key anatomical adaptive response of plants to waterlogging. Consistent with these results, waterlogging enhanced SA content in the root via expression of specific *isochorismate synthase* (*ICS*; *ICS1* and *ICS2*) and *phenylalanine ammonia lyase* (*PAL*; *PAL4*, *PAL5* and *PAL6*) genes and in the stem nodes via expression of specific *PAL* (*PAL5* and *PAL6*) genes. Although not to the same level observed in waterlogged plants, exogenous SA also induced aerenchyma formation in non-waterlogged plants. The findings of this study furthermore indicated that inhibition of ethylene synthesis in SA treated non-waterlogged and waterlogged plants does not have any effect on SA-induced emergence of axile and/or surface adventitious roots but represses SA-mediated induction of aerenchyma formation. These results highlight that the role of SA in promoting the development of axile and surface adventitious roots in waterlogged wheat plants is ethylene independent while the induction of aerenchyma formation by SA requires the presence of ethylene.

## 1. Introduction

Wheat (*Triticum aestivum* L.) is an important cereal crops world-wide; however, its production is adversely affected by several abiotic stress factors including waterlogging, which causes approximately 15–20% of the yield loss in annual wheat crop production [1]. Waterlogging significantly reduces the diffusion rate of oxygen through the soil, leading to the occurrence of hypoxic or anoxic conditions [2]. To cope with waterlogging-induced oxygen deficient conditions, plants develop several morphological and anatomical adaptive responses such as the formation of adventitious roots and root aerenchyma that improve oxygen availability to the roots [3]. Waterlogging induced development of adventitious roots in upland cereal crops such as wheat is often associated with aerenchyma formation [4,5].

Salicylic acid (SA) is one of the plant hormones that influences several plant physiological processes and mediates plant responses to a number of biotic and abiotic stresses [6,7]. With respect to abiotic stresses, SA plays an important role in mitigating the adverse effects of drought, cold, heat and salinity in many plant species including wheat [6,8]. Studies on Arabidopsis and mung bean have demonstrated the role of SA in the formation of adventitious roots under normal growing conditions [9,10,11]. For example, SA-deficient mutants of Arabidopsis *eds5-1* and *eds5-2* exhibit significantly reduced number of adventitious roots as compared to the corresponding wild type plants [11]. Further studies on mung bean revealed that induction of adventitious root formation by salicylic acid is closely associated with H_2_O_2_ accumulation. Consistently, treatment of mung bean hypocotyls with H_2_O_2_ scavenger/H_2_O_2_ biosynthesis inhibitor leads to inhibition of the SA-induced adventitious root formation [9,10]. In wheat, exogenous application of SA and its synthetic derivative, acetyl SA, has also been shown to have positive effects on several morphological traits in the shoot system including plant height, number of tillers, flag leaf area, number of spikes per plant and number of grains per spike [12,13]. 

Previous studies have also provided important insights into the roles of other plant hormones such as ethylene, auxin and cytokinin in the development of adventitious roots under normal or waterlogging conditions, and the roles of plant hormones in regulating the formation of adventitious roots under normal or waterlogging conditions often involves their synergistic/antagonistic interactions [4,14,15,16,17]. For example, the role of salicylic acid in inducing the formation of adventitious roots under normal conditions has been shown to be mediated by the modulation of auxin biosynthesis and transport [18,19]. Similarly, ethylene enhances the development of adventitious roots in waterlogged and non-waterlogged plants of different species through inducing auxin synthesis and transport, and endogenous nitric oxide levels, respectively [14,16,17], whereas cytokinin inhibits the formation of such roots via repressing auxin influx (LAX3)/efflux (PIN1) carriers [20]. A number of studies also implicate the significance of gibberellins and abscisic acid (ABA) in the regulation of adventitious root formation under waterlogged/flooded conditions [4,15,21]. Jasmonates have been reported to regulate adventitious root formation under normal conditions [22], however, our previous study suggest that this phytohormone does not play the same role under waterlogged conditions [4]. Despite these reports, the role of SA in regulating the development of such morphological adaptive responses under waterlogging conditions is poorly understood. 

The formation of root aerenchyma, an important anatomical adaptive response under oxygen-deficient conditions, has also been reported to be regulated by plant hormones mainly by ethylene via programmed death of root cortical cells [23,24]. It has been shown previously that ABA suppresses the formation of this anatomical trait under waterlogged conditions, for example, through inhibiting the elongation of phellogen-derived cells [25]. However, if the other plant hormones such as salicylic acid influence the formation of aerenchyma has not been studied.

To gain insights into the role of SA in mediating morpho-anatomical adaptations to waterlogging, this study examined the effect of exogenous SA on the formation of adventitious roots and root aerenchyma in waterlogged wheat plants, and also investigated changes in the expression patterns of SA biosynthesis genes and endogenous SA levels in the root, and basal stem nodes that produce adventitious roots in response to waterlogging.

## 2. Results

### 2.1. Salicylic Acid Induced Changes in Morpho-Anatomical Adaptive Responses to Waterlogging 

#### 2.1.1. Shoot Related Morphological Changes 

**Figure 1:**
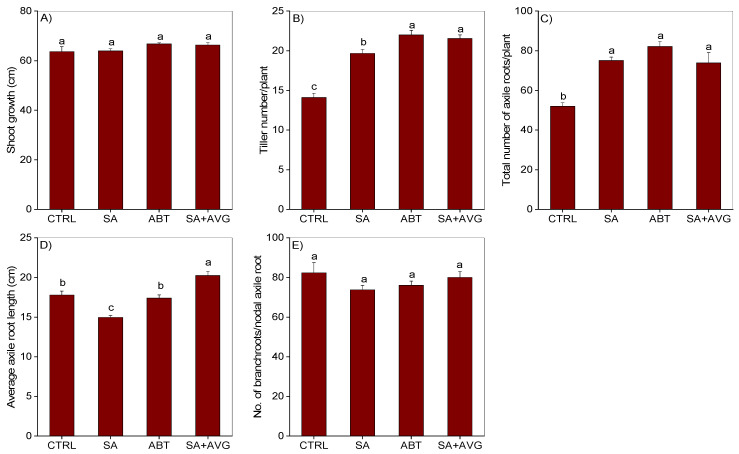
Root and shoot phenotype of non-waterlogged plants. Shoot growth (**A**) and tiller number (**B**), and total number of axile roots per plant (**C**), average axile root length (**D**), and number of branch roots per nodal axile root (**E**) in non-waterlogged plants treated with or without hormone and/or inhibitor. Data are means of three independent biological replicates (shoots and roots of at least three individual plants per replicate) ± SE. Different letters between any two samples show significant difference (one-way ANOVA; *p* < 0.05, Fisher’s LSD test). CTRL, non-waterlogged untreated; SA, salicylic acid; ABT, 1-aminobenzotriazole (SA biosynthesis inhibitor); AVG, aminoethoxyvinylglycine (ethylene biosynthesis inhibitor).

**Figure 2:**
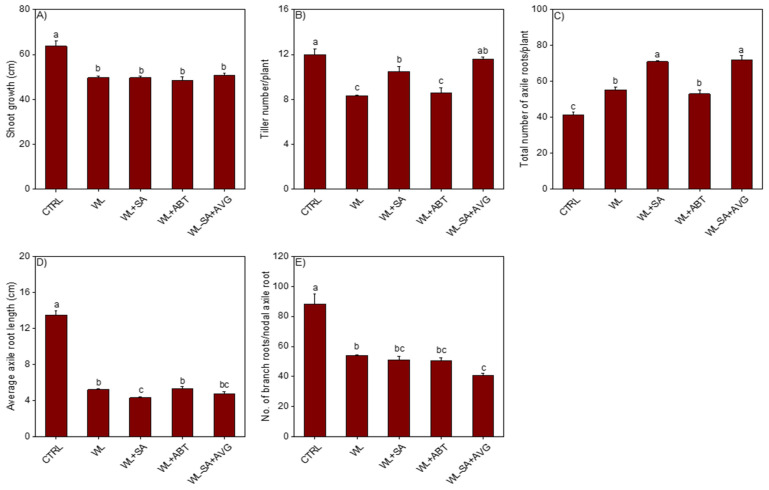
Root and shoot phenotype of waterlogged plants. Shoot growth (**A**) and tiller number (**B**), and total number of axile roots per plant (**C**), average axile root length (**D**), and number of branch roots per nodal axile root (**E**) in control non-waterlogged plants, and those waterlogged for 14 days and treated with or without hormone and/or inhibitor. Data are means of three independent biological replicates (shoots and roots of at least three individual plants per replicate) ± SE. Different letters between any two samples show significant difference (two-way ANOVA; *p* < 0.05, Fisher’s LSD test). CTRL, non-waterlogged untreated; WL, waterlogged untreated; SA, salicylic acid; ABT, 1-aminobenzotriazole (SA biosynthesis inhibitor); AVG, aminoethoxyvinylglycine (ethylene biosynthesis inhibitor).

Treatments of the non-waterlogged plants with SA or SA biosynthesis inhibitor, 1-aminobenzotriazole (ABT), significantly increased the number of tillers but did not affect shoot growth as compared to that observed in the corresponding untreated plants (Figure 1A,B). Waterlogging for 14 days significantly reduced shoot growth and tiller number relative to that observed in the non-waterlogged controls (Figure 2A,B). Treatment of the waterlogged plants with SA induced a slight but significant increase in tiller number (1.26 fold) as compared to that observed in the waterlogged untreated plants while treatment with ABT did not have any effect. Shoot growth in the waterlogged plants was not affected by treatment with SA or ABT. Since the experiments involving treatment of the non-waterlogged and 14-day waterlogged plants with or without hormone and/or inhibitor were performed at different times, the samples of the two experiments can’t be compared directly.

#### 2.1.2. Root Related Morphological Changes 

**Figure 3:**
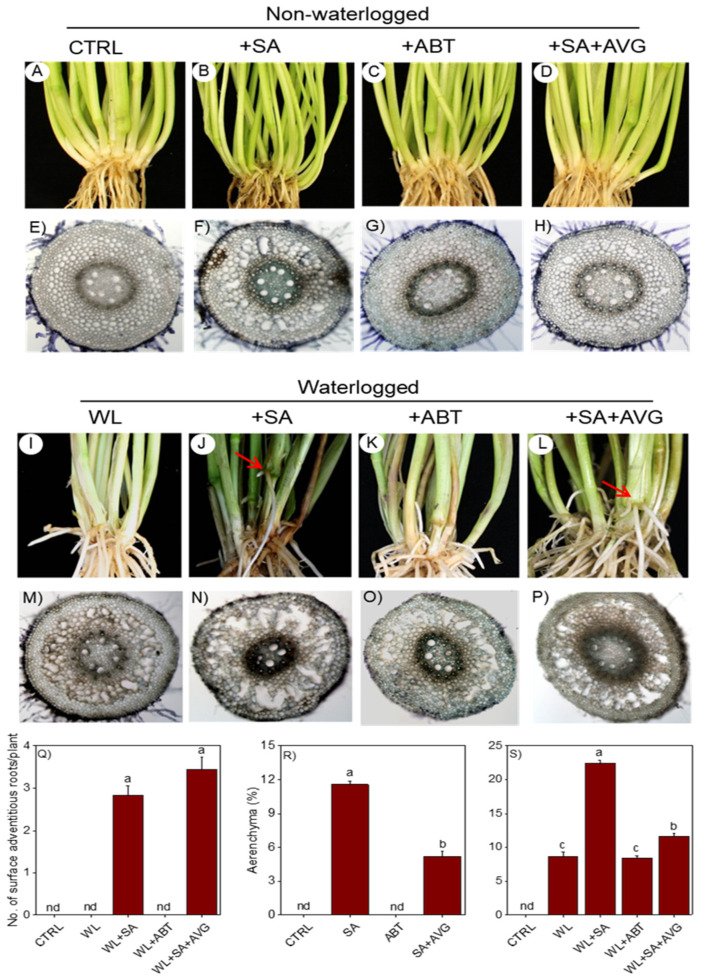
Surface adventitious root and aerenchyma formation. Development of surface adventitious roots (**A**–**D**,**I**–**L**), transverse sections of nodal roots (4 cm from the root apex) (**E**–**H**,**M**–**P**), number of surface adventitious roots (**Q**) and percentage of aerenchyma in root cross-sections (**R**,**S**) in non-waterlogged and 14-day waterlogged plants treated with or without hormone and/or inhibitor. Data are means of three independent biological replicates ± SE. Different letters between any two samples show significant difference (two-way ANOVA; *p* < 0.05, Fisher’s LSD test). Arrows indicate surface adventitious roots emergence from stem nodes. CTRL, non-waterlogged untreated; WL, waterlogged untreated; SA, salicylic acid; ABT, 1-aminobenzotriazole (SA biosynthesis inhibitor); AVG, aminoethoxyvinylglycine (ethylene biosynthesis inhibitor); nd, not detected. Hormone and/or inhibitor treatments of non-waterlogged control plants did not induce surface adventitious roots.

Treatment of the non-waterlogged plants with SA or ABT led to a similar increase in the total number of axile (primary and nodal) roots as compared to the corresponding untreated plants (Figure 1C). While treatment of the same non-waterlogged plants with SA caused a slight but significant reduction (1.23-fold) of axile root length (Figure 1D), treatment with ABT did not exert any effect on axile root length and number of branch roots per nodal axile root (Figure 1D,E). Waterlogging led to a significant increase in the total number of axile roots (1.34-fold) as compared to that observed in the non-waterlogged controls (Figure 2C). Treatment of waterlogged plants with SA caused further increase in total number of axile roots (1.72-fold) while treatment with ABT did not have any effect. In contrast, waterlogging caused significant reduction in the length of axile roots (2.58-fold) and number of branch roots per nodal axile root (1.63-fold) (Figure 2D,E). Treatment of waterlogged plants with SA or ABT did not have significant effect on the length of axile roots and number of branch roots per nodal axile root expect that SA treatment caused a slight but significant reduction (1.21-fold) in the length of axile roots. No emergence of surface adventitious roots from the basal stem nodes was evident in the non-waterlogged plants treated with/without SA or ABT, and the waterlogged plants treated with/without the SA biosynthesis inhibitor ABT (Figure 3A–C,I,K,Q). However, treatment of the waterlogged plants with SA induced the formation of surface adventitious roots (Figure 3J,Q).

#### 2.1.3. Root Anatomical Changes

Treatment of the non-waterlogged plants with SA but not ABT induced aerenchyma formation in roots (Figure 3F,G,R). Waterlogging for 14 days also induced the formation of aerenchyma as compared to that observed in the non-waterlogged controls (Figure 3S). Treatment of the waterlogged plants with SA led to a further increase (over 2.5-fold) in the percentage of aerenchyma occupying the root cross section while treatment with ABT did not exert any effect.

#### 2.1.4. Effect of Modulating Ethylene Level on SA-Induced Morpho-Anatomical Changes

Given that ethylene is a key regulator of the formation of aerenchyma and adventitious/axile roots in response to waterlogging, we treated waterlogged plants with a combination of SA and the ethylene biosynthesis inhibitor AVG to investigate if the role of SA in regulating these adaptive morpho-anatomical traits is ethylene dependent. Our data showed that AVG mediated inhibition of ethylene synthesis in the SA treated non-waterlogged and waterlogged plants did not have any apparent effect on shoot growth, axile root length and number of branch roots per nodal axile root except that it caused a slight increase (1.40-fold) of axile root length in the SA-treated non-waterlogged plants (Figure 1A,D,E and Figure 2A,D,E). Furthermore, SA-induced formation of tillers and axile roots and/or development of surface adventitious roots in the non-waterlogged or waterlogged plants were not affected by inhibition of ethylene synthesis, except that a slight but significant increase (1.10-fold) in tiller number was observed in the non-waterlogged plants (Figure 1B,C, Figure 2B,C and Figure 3Q). Inhibiting ethylene synthesis in waterlogged plants treated with SA significantly reduced the percentage of aerenchyma, however these plants still exhibited higher percentage of aerenchyma in their root cross sections than those treated with ABT or the untreated waterlogged controls (Figure 3M–P,S). Inhibiting ethylene synthesis in SA treated non-waterlogged plants also led to a decrease in the percentage of aerenchyma in the root (Figure 3E–H,R).

### 2.2. Transcriptional Regulation of Salicylic Acid Metabolism 

The biosynthesis of SA in plants has been proposed to take place through two pathways; from cinnamate via the action of phenylalanine ammonia lyase (PAL) and from chorismate through the action of isochorismate synthase (ICS) [26]. This study therefore analyzed the expression pattern of *ICS* (*ICS1* and *ICS2*) and *PAL* (*PAL4*, *PAL5* and *PAL6*) genes and SA levels in root and stem node tissues.

#### 2.2.1. Root

**Figure 4:**
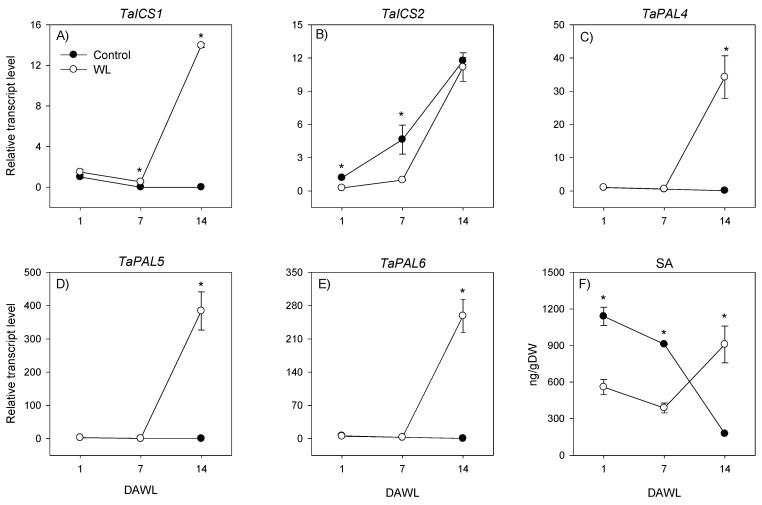
Expression of salicylic acid biosynthesis genes and salicylic acid level in roots. Relative transcript levels of the *TaICS*s (**A**,**B**) and *TaPAL*s (**C**–**E**), and salicylic acid level (**F**) in roots at 1, 7 and 14 days after waterlogging (DAWL). Transcript levels of the *TaICS*s and *TaPAL*s were determined using *Ta18SrRNA* as the reference gene and expressed relative to the transcript levels of *TaICS1* and *TaPAL4* in control roots at 1 DAWL, respectively, which were arbitrarily set a value of 1. Data are means of three independent biological replicates ± SE. Asterisks show significant difference in transcript levels between the control (non-waterlogged) and waterlogged samples within waterlogging duration (one-way ANOVA; *p* < 0.05, Fisher’s LSD test).

The expression level of *TaICS1* in the root decreased below detectable level under control condition but increased markedly in response to waterlogging for 14 days (Figure 4A). In contrast, the expression level of *TaICS2* increased in roots under both control and waterlogged conditions, however, its expression level in roots waterlogged for 1 and 7 days was over 4-fold lower than the control roots (Figure 4B). The expression levels of *TaPAL*s in control roots decreased (over 10-fold) to very low levels during the course of the waterlogging experiment (Figure 4C–E). As the waterlogging duration progressed from 1 to 7 days, the expression levels of *TaPAL*s in waterlogged roots also decreased (over 1.7-fold). However, waterlogging for 14 days resulted in substantial inductions (over 33-fold) in the expression levels of *TaPAL*s, leading to the observation of their higher expression levels (over 450-fold) than that observed in the control roots.

The levels of endogenous SA in both control and waterlogged roots exhibited slight decreases as the duration of waterlogging progressed from 1 to 7 days (Figure 4F). However, from 7 to 14 days after waterlogging (DAWL) the root SA level in waterlogged roots exhibited an increase (2.3-fold) while it decreased further (over 5-fold) in the corresponding non-waterlogged control roots. Relative to the SA level detected in the control roots, waterlogged roots exhibited ≥2-fold lower SA level at 1 and 7 DAWL but 5-fold higher SA level at 14 DAWL.

#### 2.2.2. Stem Node

**Figure 5:**
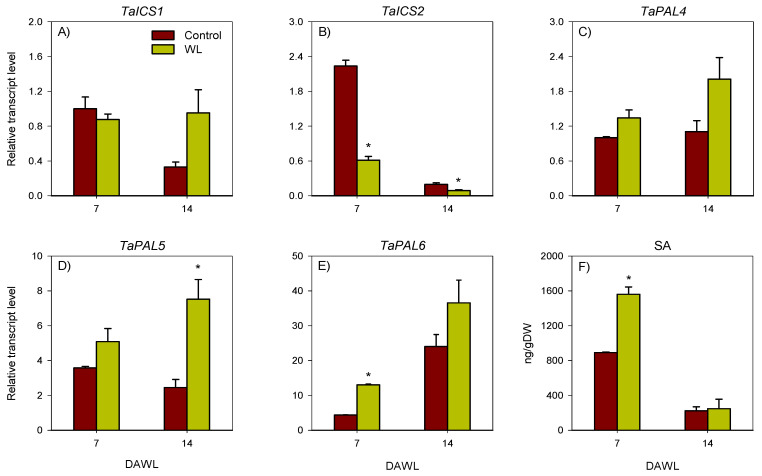
Expression of salicylic acid biosynthesis genes and salicylic acid level in stem nodes. Relative transcript levels of the *TaICS*s (**A**,**B**) and *TaPAL*s (**C**–**E**), and salicylic acid level (**F**) in stem nodes at 7 and 14 days after waterlogging (DAWL). Transcript levels of the *TaICS*s and *TaPAL*s were determined using *Ta18SrRNA* as the reference gene and expressed relative to the transcript levels of *TaICS1* and *TaPAL4* in control stem nodes at 7 DAWL, respectively, which were arbitrarily set a value of 1. Data are means of three independent biological replicates ± SE. Asterisks show significant difference in transcript levels between the control (non-waterlogged) and waterlogged samples within waterlogging duration (one-way ANOVA; *p* < 0.05, Fisher’s LSD test).

As compared to that detected at 7 DAWL, the expression level of *TaICS1* in the control stem nodes decreased by 14 DAWL while it remained unaffected in the corresponding samples collected from waterlogged plants (Figure 5A). Irrespective of this change, no significant difference in the expression level of *TaICS1* was observed between the two stem node samples at both time points. The expression level of *TaICS2* observed at 7 DAWL decreased substantially in both control and waterlogged stem nodes by 14 DAWL (Figure 5B). However, waterlogging for either 7 or 14 days led to reduction (over 2-fold) in its expression level relative to the respective controls. The expression levels of *TaPAL*s in both control and waterlogged stem nodes was not affected or increased slightly as the waterlogging duration progressed from 7 to 14 days except that the expression level of *TaPAL6* exhibited marked increase in both samples (Figure 5C–E). When the expression levels of *TaPAL*s were compared between the two stem node samples, the waterlogged samples exhibited significantly higher expression level of *TaPAL5* (over 3-fold fold) and *TaPAL6* (3-fold) at 14 and 7 DAWL, respectively. 

Irrespective of growth conditions, the stem node SA levels detected at 7 DAWL exhibited marked reductions by 14 DAWL (Figure 5F). Stem nodes collected from waterlogged plants exhibited higher level of SA (1.8-fold) than the respective controls only at 7 DAWL. 

## 3. Discussion

Plant hormones such as ethylene and auxin play important roles in mediating plant adaptation to waterlogging/oxygen deficient conditions [4,14,15,16,23]. This study investigated the role of SA in regulating morpho-anatomical adaptive responses to waterlogging in wheat. The observation of root morphological changes in response to waterlogging including enhanced formation of axile roots, and inhibition of axile root elongation and lateral roots emergence is consistent with previous reports [4,16,23,27]. The association of these alterations in root morphological traits due to waterlogging with upregulation of specific root SA biosynthesis genes and induction of root SA content implies the significance of SA in forming a shallow root system and thereby tolerance to waterlogging conditions. However, as compared to the corresponding non-waterlogged plants, lower root SA level was evident in plants waterlogged for 1 and 7 days. Given that waterlogging induces accumulation of ethylene [23], which has been reported to suppress SA biosynthesis [28], the observed decrease in SA level might imply the responsiveness of SA biosynthesis to ethylene during the initial phases of waterlogging.

It has been shown previously that exogenous SA induces the development of adventitious roots in several plant species grown under normal aerated conditions [9,10]. This effect of SA is reported to be associated with the activation of the de novo synthesis of auxin, which is known to play critical role in the formation of adventitious roots [18], and its rootward transport and the repression of its shootward and non-polar transport in the root tips [19]. Consistent with these results, exogenous SA promoted the formation of axile roots in our non-waterlogged wheat plants. However, the induction of axile root formation in non-waterlogged plants was also prevalent in response to inhibition of SA, implying that the development of such roots is not entirely dependent on SA. The reduction of axile root length in the non-waterlogged wheat plants by SA along with its induction via inhibition of ethylene synthesis in the SA treated plants indicates the significance of ethylene in controlling the elongation of axile root cells. Consistently, ethylene has been shown to inhibit root growth in Arabidopsis plants grown under aerated condition through repressing cell elongation, which is mediated by its effect on local auxin biosynthesis and transport [29]. The observation of further induction in the formation of axile roots and reduction of root elongation in waterlogged plants due to exogenous SA indicates the importance of SA in promoting these root morphological traits and enhance adaptation to waterlogging. This result is in agreement with a previous report that showed the presence of higher SA level and development of more adventitious roots in waterlogging tolerant than waterlogging sensitive soybean lines grown under waterlogged condition [30]. Furthermore, exogenous SA has been shown to inhibit primary root elongation and lateral root formation but enhance adventitious root development, leading to the formation of a shallow root system in Arabidopsis [19]. The role of SA in suppressing primary root elongation and lateral root organogenesis has been shown to be associated with its negative effect on auxin transport through inhibiting protein phosphatase 2A, which regulates the activity of PIN auxin efflux carriers [31]. 

Despite inductions in the expression levels of stem node *TaPAL5* and *TaPAL6* genes and the level of SA in response to waterlogging for 7 days, no emergence of surface adventitious roots from the stem nodes was apparent at 14 DAWL. However, exogenous SA was able to promote the formation of these roots from the basal stem nodes only in wheat plants waterlogged for 14 days. Given that SA has been shown to induce adventitious root formation in a concentration dependent manner [9], our data suggest that the amount of SA produced by the basal stem nodes of untreated waterlogged plants was not sufficient to trigger this adaptive response. It has been shown previously that SA enhances adventitious root formation in Arabidopsis plants grown under aerated conditions through regulating the biosynthesis and transport of auxin [19], which has also been implicated in the regulation of redifferentiation of stem cells to form adventitious root primordia [18]. For instance, exogenous SA has been shown to induce the accumulation of free IAA and formation of adventitious roots in cucumber hypocotyls, and this induction of IAA accumulation by SA has been reported to be mediated by inhibition of IAA conjugation via competitive repression of the activity of auxin conjugating enzyme Gretchen Hagen3.5 [32]. On the other hand, treatment of cucumber hypocotyls with auxin transport inhibitor 1-naphthylphthalamic acid has been shown to completely inhibit SA-induced adventitious root formation. It is therefore likely that the exogenous SA applied to waterlogged wheat plants was able to induce auxin level and transport, and thereby trigger the formation of surface adventitious roots. In support of this, a close association between an increase in the level of auxin in the basal stem nodes and induction of surface adventitious root emergence has been observed in wheat plants waterlogged for durations longer than those considered in this study [4]. It is well established that ethylene is a key signaling molecule in promoting adventitious root formation in waterlogged plants including wheat [4,14,15,16]. The absence of any effect of inhibition of ET synthesis in non-waterlogged or waterlogged wheat plants treated with SA on SA-induced increases in the number of axile and/or surface adventitious roots indicate that SA regulates the formation of these roots independent of ethylene. 

The prevalence of enhanced aerenchyma formation in the roots of both waterlogged and non-waterlogged wheat plants in response to exogenous SA, although its effect was more pronounced in waterlogged plants, indicates the role of SA in inducing this anatomical adaptive trait. Aerenchyma development, which facilitates oxygen transport and availability in waterlogged roots, is controlled mainly by ethylene [23,24]. Although the formation of aerenchyma in dry land plant species is uncommon under well-drained/aerated soil conditions, previous studies have reported that exogenous ethylene can promote the development of this anatomical trait in the roots of such plant species including wheat and maize when grown under aerated conditions [23,33]. Therefore, the repression of SA-induced aerenchyma formation in both non-waterlogged and waterlogged plants due to inhibition of ethylene synthesis suggests that the induction of aerenchyma development by SA is dependent at least partly on ethylene. In agreement with this, enhancement of aerenchyma formation in waterlogging tolerant as compared to waterlogging sensitive soybean lines grown under waterlogged condition has been shown to be associated with increases in the levels of both SA and ethylene but not that of ethylene alone [30].

The absence of any effect of exogenous SA on shoot growth under both non-waterlogged and waterlogged conditions suggests that SA does not have primary role in the regulation of shoot growth in wheat plants. In contrast, treatment with SA induced tiller formation under both conditions. The formation of tillers from axillary meristem is regulated by phytohormones such as cytokinin (CK) that acts as positive regulator of tillering, and auxin and strigolactones (SL), which act as inhibitors of tillering [34]. It has been shown previously that SA induces the expression levels of CK signaling genes such as those encoding the Arabidopsis histidine phosphotransfer (*AHP6*), which acts as positive regulator of CK signaling [35], and the Arabidopsis response regulator (*ARR*), which is a known mediator of CK signaling [36]. Furthermore, induction of tillering in tobacco plants overexpressing *Ras-related small GTP-binding protein 1* (*RGP1*) gene has been shown to be associated with increases in the levels of both CK and SA [37]. It therefore likely that the exogenous SA applied to our wheat plants induced CK production and signaling, and thereby tiller initiation. Previous reports have also implicated SA in the regulation of the biosynthesis, transport and signaling of auxin, which acts as important regulator of tiller formation/branching in plants. For example, SA represses the expression of genes encoding auxin receptor (*TIR1*) and auxin importer and exporter (*AUX1* and *PIN7*) [38], and such suppression of auxin transport and signaling has been shown to lead to an increase in the number of tillers [39,40]. Therefore, the prevalence of enhanced tiller formation in response to SA might suggest the suppression of auxin production/signaling and its transport. Previous studies have also demonstrated the interaction between SA and strigolactone (SL), a phytohormone that plays an important role in the regulation of tillering. For instance, treatment of Arabidopsis seedlings with exogenous GR24, synthetic analogue of SL, induces SA level while SA level has been shown to be suppressed in *more axillary growth2* (*max2*) mutant plants that are defective in SL signaling [41]. Therefore, the observation of induction in tiller formation in response to inhibition of SA biosynthesis in the non-waterlogged wheat plants might imply suppression of SL signaling and thereby an increase in tiller number.

**Figure 6:**
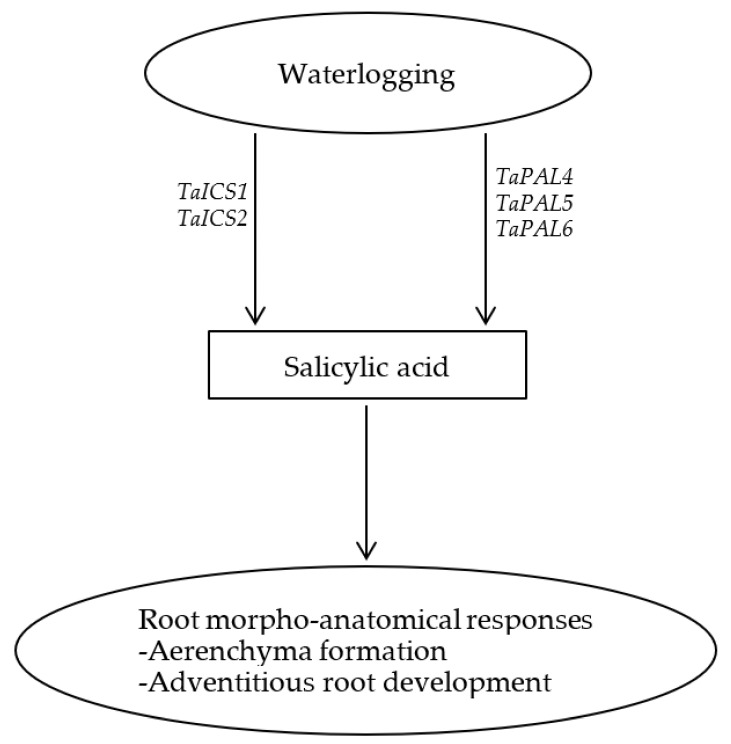
Schematic representation of the role of salicylic acid in inducing aerenchyma formation and development of adventitious roots in wheat under waterlogging condition.

In summary, our data showed that SA enhances the development of axile and surface adventitious roots in waterlogged wheat plants but represses their elongation, leading to the formation of shallow root system. This along with the pronounced induction of root aerenchyma formation by SA highlights the significance of SA in mediating morpho-anatomical responses and thereby adaptation of wheat plants to waterlogging as depicted in Figure 6.

## 4. Materials and Methods

### 4.1. Plant Material and Growth Conditions

Hexaploid wheat cv. Harvest was used in this study. Growing the experimental plants and subjecting them to waterlogging treatments were performed exactly as described in Nguyen et al. [6]. Mature dry seeds were surface sterilized and germinated in a Petri-plate system for 3 days and then the germinated seeds were transplanted into 3 L plastic pots containing top soil and sand (2:1, *v*:*v*) supplied with 18 g of fertilizer (ACER^®^nt 13-12-12 consisting of 13% N, 12% P_2_O_5_, 12% K_2_O and micro elements). Plants were grown in a growth room under 22/20 °C (day/night) and 16/8 h photoperiod condition. Thirty days old plants were subjected to waterlogging treatment. To this end, each pot containing the plants were submerged in a 6 L pot filled with water and the water level was maintained at ~2 cm above the surface of the soil during the entire period of the experiment by adding more water as needed. The respective non-waterlogged plants were placed in 6 L pots with no water, and the plants were watered with ~0.5 L/pot once every two days throughout the experimental period. Roots were collected from waterlogged and respective control plants at 1, 7 and 14 days after the start of waterlogging (DAWL), while basal stem nodes were collected only from plants waterlogged for 7 and 14 days and their respective controls since they were not developed in the main stems/tillers of most plants waterlogged only for 1 day. Separation of the roots from the soil and harvesting of the basal stem nodes were performed as described previously [6]. In brief, pots containing the plants were placed in a plastic box (~85 L) filled with room temperature tap water, and the plants along with the soil were first separated from the pot and then the soil was carefully removed from the roots. Finally, the roots were washed with a gently running tap water to remove any remaining soil. Tissue samples (three independent biological replicates) designated for gene expression and SA level analyses were frozen in liquid nitrogen immediately after harvest and then stored at −80 °C until further use. Freshly harvested tissues were used for examining the anatomical and morphological parameters considered in the study.

### 4.2. Pharmacological Treatments

Aerial parts of the plants waterlogged for 14 days and their corresponding non-waterlogged plants were sprayed (10 mL/plant) with 1 mM of SA (Tokyo Chemical Industry, Tokyo, Japan) or 100 µM of 1-aminobenzotriazole (ABT; SA biosynthesis inhibitor) (Tokyo Chemical Industry) or 100 µM aminoethoxyvinylglycine (AVG; ethylene biosynthesis inhibitor) (Cayman Chemical, Ann Arbor, MI, USA) or combination of SA (1 mM) plus AVG (100 µM) in 0.1% (*v*/*v*) aqueous Tween-20 at 1 day before the start of waterlogging and at 3 and 7 DAWL. Non-waterlogged and waterlogged untreated control plants were sprayed with 0.1% (*v*/*v*) aqueous Tween-20. 

### 4.3. Morphological and Anatomical Examination of Roots 

The length and number of axile roots (primary roots and nodal adventitious roots that originate below soil surface), number of branch/lateral roots that originate from axile roots, number of axile roots that originate from basal stem nodes (hereafter referred to as surface adventitious roots), shoot length and tiller number were recorded (three individual plants per replicate with a total of three replicates) as described in Nguyen et al. [6]. 

Root cross sections were prepared from the nodal root segments (at 4 cm from the root apex) harvested from 14-day waterlogged and the corresponding non-waterlogged wheat plants treated with or without hormone and/or inhibitor. Cross-sections were prepared using a razor blade and then they were stained in 0.05% (*w*/*v*) toluidine blue O in benzoate buffer (pH 4.4) for 2 min. Subsequently, root sections were destained and analyzed using trinocular compound epi-fluorescence microscope fitted with digital camera (OMAX, China). The percentage of root cross section covered by aerenchyma was determined using ImageJ software version 1.53e as described in Haque et al. [42].

### 4.4. Identification of SA Metabolic Genes

Identification of the SA metabolism genes, naming of the genes/homologs, designing of the respective primers and confirmation of their gene specificity were performed as described in Nguyen et al. [6]. Primer information of the targeted genes is shown in Table S1.

### 4.5. RNA Extraction and qRT-PCR Analysis

Total RNA from the root and basal stem node tissues was extracted using TRIzol reagent (Thermo Fisher Scientific, Waltham, MA, USA). DNase treatment of the total RNA and subsequent cDNA synthesis and qPCR assays were performed as described previously [43]. Relative gene expression levels were determined according to Livak and Schmittgen [44] after normalization of the qPCR data using *Ta18SrRNA* as a reference gene. qRT-PCR efficiency of the genes was determined as described in Yao et al. [45].

### 4.6. Measurement of Endogenous SA Content

Extraction and subsequent purification of SA from the tissue samples was conducted as described previously [46] except that 10 mL of 80% (*v*/*v*) acetonitrile containing 1% (*v*/*v*) acetic acid was used for the extraction procedure. Analysis of SA level was performed using LC-ESI-MS/MS (Agilent 1260–6430; Agilent, Santa Clara, CA, USA) as described in Yoshimoto et al. [47].

### 4.7. Statistical Analysis 

Statistically significant difference between samples was determined using either one-way or two-way ANOVA. Comparison of sample means was carried out using Fisher’s least significance difference (LSD) test at *p* < 0.05. 

## Data Availability

Not applicable.

## Supplementary Materials

The following supporting information can be downloaded at https://www.mdpi.com/article/10.3390/ijms23031243/s1.
